# Improved effect of fresh ginseng paste (radix ginseng-ziziphus jujube) on hyperuricemia based on network pharmacology and molecular docking

**DOI:** 10.3389/fphar.2022.955219

**Published:** 2022-10-25

**Authors:** Hao Zhang, Wei Liu, Si-Min Qi, Jian-Feng Chi, Qiang Gao, Xiang-Hui Lin, Shen Ren, Zi Wang, Xiu-juan Lei, Wei Li

**Affiliations:** ^1^ College of Chinese Medicinal Materials, National and Local Joint Engineering Research Center for Ginseng Breeding and Development, Jilin Agricultural University, Changchun, China; ^2^ College of Life Sciences, Engineering Research Center of the Chinese Ministry of Education for Bioreactor and Pharmaceutical Development, Jilin Agricultural University, Changchun, China; ^3^ Liaoning Xifeng Pharmaceutical Group Co., Ltd., Huanren, China

**Keywords:** radix ginseng, ziziphus jujube, fresh ginseng paste, hyperuricemia, network pharmacology, molecular docking

## Abstract

**Background:** Hyperuricemia (HUA) is a metabolic disease caused by reduced excretion or increased production of uric acid. This research aims to study the practical components, active targets, and potential mechanism of the “Radix ginseng (RG)-Ziziphus jujube (ZJ)” herb pair through molecular docking, network pharmacology, and animal experiments.

**Methods:** The potential targets of “Radix ginseng (RG)-Ziziphus jujube (ZJ)” herb pair were obtained from the TCMSP database. The therapeutic targets of HUA were acquired from the GendCards, OMIM, PharmGkb, and TTD databases. Protein-protein interaction network (PPI) was constructed in the STRING 11.0 database. The David database was used for enrichment analysis. Molecular Docking was finished by the AutoDock Vina. And we employed Radix ginseng and Ziziphus jujube as raw materials, which would develop a new functional food fresh ginseng paste (FGP) after boiling. In addition, benzbromarone (Ben) (7.8 mg/kg) and allopurinol (All) (5 mg/kg) were used as positive drugs to evaluate the hyperuricemia induced by FGP (400 and 800 mg/kg) potassium oxazine (PO) (100 mg/kg) and hypoxanthine (HX) (500 mg/kg) on mice.

**Results:** The results showed that 25 targets in the “RG-ZJ” herb pair interacted with hyperuricemia. Then protein-protein interaction (PPI) analysis showed that TNF, IL-1β, and VEGFA were core genes. KEGG enrichment analysis showed that the Toll-like receptor signaling pathway and IL-17 signaling pathway were mainly involved. Meantime, animal experiments showed that FGP could improve the HUA status of mice by reducing serum UA BUN, XO, and liver XO levels (*p* < 0.05, *p* < 0.01). Furthermore, we analyzed the main ingredients of FGP by HPLC. We found that the main ingredients of FGP had solid binding activity to the core target of HUA by molecular docking.

**Conclusion:** This study explored the active ingredients and targets of the “RG-ZJ” herb pair on HUA through network pharmacology, molecular docking, and animal experiments. It revealed the improvement of FGP in mice with HUA.

## Introduction

Hyperuricemia (HUA) is a disease caused by purine metabolic disorder or decreased uric acid (UA) excretion leading to increased serum UA levels (Wu et al., 2020). At present, people’s living standards continue to improve, and the diet structure has undergone unreasonable changes. People’s excessive intake of high protein and purine food leads to an increasing trend in the number of HUA patients, characterized by younger age (Simin et al., 2022). HUA can cause gout, also an inducing factor for hypertension, obesity, cardiovascular disease, renal failure, and other diseases (Ma et al., 2019; Bezerra et al., 2021; Yang et al., 2022). However, the current first-line drugs to treat HUA have specific side effects, such as Benzbromarone (Dalbeth et al., 2021) and Allopurinol (Khanna et al., 2012).

In the development process of traditional Chinese medicine (TCM), herb pairs (mixture of two herbs), as the basic composition units of Chinese herbal formulas, have special clinical significance in TCM and are much simpler than other complex formulas without altering their basic therapeutic features (Han et al., 2016). Radix ginseng (RG) contains various active ingredients such as ginsenosides and polysaccharides. It has various pharmacological activities, such as pharmacological activities of antibiosis, anti-tumor activities, antioxidation, anti-diabetes, and anti-cardiovascular diseases (Khanna et al., 2012; Li et al., 2015). Moreover, it has strong liver and kidney protection. In addition, many studies have shown that the secondary saponins in ginseng have better pharmacological activities, such as ginsenoside Rk3, Rh4, Rg3, Rg5, and Rh2 (Yu et al., 2012; Li et al., 2016; Zhang et al., 2018; Hu et al., 2019; Liu et al., 2020). Ziziphus jujube (ZJ) is a healthy food containing various biologically active substances, such as polysaccharides, polyphenols, alkaloids, and other nutrients (Hernández et al., 2016; Lu et al., 2021). These nutrients from ZJ have physiological functions, including antioxidant (Zhao et al., 2014), anti-inflammatory, hypoglycemia (Kawabata et al., 2017), and anti-hyperlipidemia (Jeong and Kim, 2019). Both RG and ZJ are typical medicinal and edible medicinal materials with good pharmacological activities. The “RG-ZJ” herb pair has greater medicinal value. In this study, we developed a potential functional food Fresh Ginseng Paste (FGP), which was prepared by cooking Radix ginseng (RG)and Ziziphus jujube (ZJ) in the best proportion. The preparation method of fresh ginseng paste is to select fresh ginseng and jujube (RG: ZJ = 1: 3). Firstly, the ginseng was purified, and the ginseng whiskers were separated from the main root. At the same time, the jujube was crushed into 200 mesh very fine powder, and then the ginseng whiskers were added to the purified water with a ratio of 1: 30 according to the mass. After the fire was boiled, the fire was boiled for 30 min. After repeating twice, it was crushed into a homogenate and mixed with the jujube very fine powder. At 45°C, the control pressure was 1.1 Mpa for 30 min, and it was boiled into a thick paste. FGP has been proved to have pharmacological activities such as promoting sleep (Su et al., 2021) and anti-fatigue and has great potential for development. Furthermore, FGP contains abundant saponins and flavonoids, which can be effectively improved.

Based on the above research, in the study, network pharmacology was employed to predict the mechanism of action of the “RG-ZJ” herb pair on HUA intervention, screen out the foremost practical components, and predict the targets that it acts on HUA. To verify the above results, the improvement effect of FGP on HUA in mice was preliminarily explored.

## Materials and methods

### Materials and equipment

Sixteen ginsenosides 20(S)-ginsengoside Rg2, 20 (S)-ginsengoside Rh1, 20 (R)-ginsenoside Rh1, F1, Rg6, F4, Rk3, Rh4, 20 (S)-sinsenoside Rg3, 20 (R)-ginsenoside Rg3, CK, Rk1, Rg5, 20 (S)-ginsenoside Rh2, 20 (R)-ginsenoside Rh2, and PPD standards were purchased from the Hongjiu Biotech Co., Ltd. (Jilin, China). The purity of all these standards was over 98% as indicated by the manufacturer. HPLC-grade acetonitrile was purchased from Merck Co. (Merck, Darmstadt, Germany). Wahaha purified water was purchased from Hangzhou Wahaha Group Co., LTD. Ultraviolet spectrophotometer (UV) was purchased from Shanghai Metash instruments Co., LTD. (UV-9000, Shanghai, China). Other chemicals were of reagent grade. Waters e 2695 HPLC system (Palo Alto, CA, United States).

### Sample preparation

Fresh Ginseng Paste (FGP) was produced and provided by Liaoning XIFENG Pharmaceutical Group Co., Ltd. (License No. SC10721052200667). FGP based on “RG-ZJ”, selected natural growth was born of *Panax ginseng* C.A. Meyer cv. Silvatica, covered for 5 years with high quality, using the unique patented technology, scientific processing, transformation of rare active substances, enrichment of the represented by Rg3, Rk1, Rg5, of 16 kinds of rare saponins group based on maximum keep the effective active substances in the ginseng. 5 g FGP sample was extracted three times with 100 ml of analytical pure methanol at room temperature by ultrasonication (60 kHz, heat power 330 W; KQ-600KDB, Kun-shan, China) for 60 min. The residue was dissolved in 1 ml of solvent (MeOH: H2O = 1:1, v/v), and then filtered through a 0.45 µm polytetrafluoroethylene (PTFE) syringe filter (Waters, Milford, MA, United States) and it was ready for HPLC analysis.

### Chromatographic conditions

Samples were analyzed on a Waters e2695 HPLC system (Waters Corporation, Milford, United States) equipped with a UV detector. Analytes were separated on a Waters C18 column (4.6 cm × 25 cm, 5 μm) at 30 °C and the detection wavelength was set at 203 nm. The mobile phase consisted of a mixture of acetonitrile (A) and water (B) and were set in a gradient elution program: 0–40 min, 18.0%–21.0% A; 40–42 min, 21.0–26.0% A; 42–46 min, 26.0%–32.0% A; 46–66 min, 32.0%–34.0% A; 66–71 min, 34.0%–38.0% A; 71–78 min, 38.0%–49.1% A; 78–82 min, 49.1% A. 82–83 min, 49.1%–50.6% A.83–88 min, 50.6%–59.6% A.88–90 min, 59.6%–65% A.90–97 min, 65.0% A. 97–102 min, 65.0%–75.0% A.102–110 min, 75.0%–85.0% with a flow rate of 1.0 ml/min. The injection volume was 20 μl.

### Determination of ginsenosides in fresh ginseng paste

20(S)-ginsengoside Rg2, 20 (S)-ginsengoside Rh1, 20 (R)-ginsenoside Rh1, F1, Rg6, F4, Rk3, Rh4, 20 (S)-sinsenoside Rg3, 20 (R)-ginsenoside Rg3, CK, Rk1, Rg5, 20 (S)-ginsenoside Rh2, 20 (R)-ginsenoside Rh2, and PPD were prepared and diluted with 20% (v/v) methanol aqueous solution to appropriate concentration for the establishment of calibration curves (Table 1). The content of total saponins in FGP was calculated by UV detection method, and the type and content of ginsenosides were calculated by normalization method of standard curve above.

**TABLE 1:** Active Ingredients of the RG- ZJ herb pair.

1	Herb	ID	Ingredients	OB%	DL
2	ZJ	MOL012921	Stepharine	31.55	0.33
3	ZJ	MOL012940	Spiradine A	113.52	0.61
4	ZJ	MOL012946	zizyphus saponin I	32.69	0.62
5	ZJ	MOL012961	jujuboside A	36.67	0.62
6	ZJ	MOL012976	coumestrol	32.49	0.34
7	ZJ	MOL012980	Daechuine S6	46.48	0.79
8	ZJ	MOL000422	kaempferol	41.88	0.24
9	ZJ	MOL012981	Daechuine S7	44.82	0.83
10	ZJ	MOL012986	Jujubesaponin V	36.99	0.63
11	ZJ	MOL012989	Jujuboside C	40.26	0.62
12	ZJ	MOL012992	Mauritine D	89.13	0.45
13	ZJ	MOL000263	Oleanolic Acid	29.028	0.26
14	ZJ	MOL001454	berberine	36.86	0.78
15	ZJ	MOL001522	(S)-Coclaurine	42.35	0.24
16	ZJ	MOL000211	Mairin	55.38	0.78
17	ZJ	MOL003410	Ziziphin	66.95	0.62
18	ZJ	MOL004350	Ruvoside	36.12	0.76
19	ZJ	MOL000492	(+)-catechin	54.83	0.24
20	ZJ	MOL000627	Stepholidine	33.11	0.54
21	ZJ	MOL007213	Nuciferin	34.43	0.4
22	ZJ	MOL000783	Protoporphyrin	30.86	0.56
23	ZJ	MOL008034	Ceanothic acid	73.52	0.77
24	ZJ	MOL008647	Moupinamide	86.71	0.26
25	ZJ	MOL002773	β-carotene	37.18	0.58
26	RG	MOL002879	Diop	43.59	0.39
27	RG	MOL000449	Stigmasterol	43.83	0.76
28	RG	MOL000358	β-sitosterol	36.91	0.75
29	RG	MOL003648	Inermin	65.83	0.54
30	RG	MOL004492	Chrysanthemaxanthin	38.72	0.58
31	RG	MOL005308	Aposiopolamine	66.65	0.22
32	RG	MOL005314	Celabenzine	101.88	0.49
33	RG	MOL005317	Deoxyharringtonine	39.27	0.81
34	RG	MOL005318	Dianthramine	40.45	0.2
35	RG	MOL005320	arachidonate	45.57	0.2
36	RG	MOL005321	Frutinone A	65.9	0.34
37	RG	MOL005344	20(S)-ginsenoside Rh2	36.32	0.56
38	RG	MOL005348	Ginsenoside-Rh4	31.11	0.78
39	RG	MOL005356	Girinimbin	61.22	0.31
40	RG	MOL005357	Gomisin B	31.99	0.83
41	RG	MOL005360	malkangunin	57.71	0.63
42	RG	MOL005376	Panaxadiol	33.09	0.79
43	RG	MOL005384	suchilactone	57.52	0.56
44	RG	MOL005399	alexandrin_qt	36.91	0.75
45	RG	MOL005401	ginsenoside Rg5	39.56	0.79
46	RG	MOL000787	Fumarine	59.26	0.83

### Chemicals and reagents

Hypoxanthine (CAS. 68-94-0, HX); Potassium oxazine (CAS. 2207-75-2, PO); Benzbromarone (CAS. 3562-84-3, Ben); Allopurinol (CAS. 315-30-0, All) (Solabor Bio Co., Ltd.); Commercial assay kits for uric acid (UA), xanthine oxidase (XO), blood urea nitrogen (BUN) and hematoxylin and eosin (H&E) dye kits were purchased from Nanjing Jiancheng Bioengineering Institute (Nanjing, China).

### Ingredients from “ RG-ZJ ” and hyperuricemia target prediction

All the ingredient data of Radix ginseng (RG) and Ziziphus jujube (ZJ) were retrieved from TCMSP (https://tcmspw.com/tcmsp.php) and Swiss target, RG and ZJ as the search keywords, oral bioavailability (OB) ≥ 30%, drug-like properties (DL) ≥ 0.18 for screening, and then target prediction. Then, the target information of hyperuricemia (HUA) was predicted through the above four databases of GendCards, OMIM, PharmGkb, and TTD, and the repeats were removed and intersected with the drug targets. In addition, the Venn diagram is used for visualization.

### Enrichment analysis and network construction

Common target proteins screened by the “RG-ZJ” herb pair and HUA were uploaded to String (https://string-db.org/, v11.0) online platform database (Chen et al., 2018; Lee et al., 2018), to construct a protein-protein interaction network model and obtain a PPI interaction network. Then the biological process (BP), molecular function (MF), cellular component (CC), and role of “RG-ZJ” in the potential targets of the HUA intervention pathway were explored. This study used the critical targets of proteins to submit the PPI network to the DAVID database (https://david.ncifcrf.gov/, v6.8) (Jiang et al., 2022), for GO (Gene Ontology) bioprocess enrichment analysis and KEGG pathway enrichment analysis.

### Animals and experimental design

6-week-old male ICR mice (weighting 18–23 g) were obtained from Liaoning Changsheng Biotechnology Co., Ltd. (Certificate No. SCXK [Liao] 2020-0001). The mice underwent adaptive feeding for at least one week. All animal handling and experimental procedures are maintained in accordance with the Ethical Committee approval for Laboratory Animals of Jilin Agricultural University.

In this study, HUA was induced in mice according to the method in the previous study [24]. All experimental animals were randomly divided into six groups (*n* = 8): normal group, HUA group, positive groups (All: 5 mg/kg/day; Ben: 7.8 mg/kg/day), and FGP treatment (400 and 800 mg/kg) groups. All groups except the normal group received intraperitoneal injection of PO (100 mg/kg/day) and gavage administration of HX (500 mg/kg/day) for seven consecutive days to elevate UA levels. The same volume of saline was employed in the normal group. The FGP group was pre-administered with 400 and 800 mg/kg and prepared FGP into suspension with normal saline once a day for seven consecutive days. From the 14th day, all groups except the normal group were injected with PO (100 mg/kg) and administered intragastrically with HX (500 mg/kg) daily. The continuous molding and administration were performed for 7 days. To ensure efficacy, Ben (7.8 mg/kg) and All (5 mg/kg) were administered by intragastric administration one day before the model was established in the positive group. In addition, we fed the normal group with physiological saline. Twelve hours after injection, blood samples were gathered and allowed to clot for 45 min at room temperature. Then, the serum was centrifuged (3500 rpm, 10 min, and 4°C) and stored at −20°C for biochemical analysis, including UA, BUN, and XO detection. The remaining serum was stored in a −80°C refrigerator for subsequent indicators detection. After weighing the anatomical liver and kidney tissues, part of them were fixed in 10% neutral formalin buffer, and part of them was wrapped with tin paper, frozen in liquid nitrogen, and stored at −80°C.

### Determination of biochemical parameters

The activities of uric acid (UA), Xanthine oxidase (XO), and blood urea nitrogen (BUN) in serum and the level of XO in liver tissues were quantified by those mentioned above in commercially available kits (Nanjing Jiancheng Bioengineering Institute, Nanjing, China) according to the manufacturer’s protocol. The absorbance was measured at the corresponding wavelength using an automatic microplate reader (Bio Tek Elx800, Berton Instruments, United States).

### hematoxylin and eosin staining

Kidney tissues were collected and then washed with phosphate-buffered saline. The tissues were fixed with 4% paraformaldehyde for 24 h, embedded in paraffin, and cut into 5 μm thickness. The histopathological changes were mounted with neutral gum, and representative images were captured using a light microscope (Olympus BX-60, Tokyo, Japan).

### Molecular docking

The binding ability of key compounds including (20R)-ginsenoside Rg3, ginsenoside Rg5, ginsenoside Rh4, oleanolic acid, and kaempferol to key targets in FGP was verified by molecular docking. The above compounds were determined in the PubChem database (https://pubchem.ncbi.nlm.nih.gov/), and the crystal structures of TNF-α-P01375, IL-1β-M4WG34, VEGFA-P15692 and XO-P80457 were obtained from the Protein Data Bank database (http://www.rcsb.org/) in PDB format. AutoDockTools-1.5.6 was used to save protein and ligand data in PDBQT format. Finally, AutoDockVina.exe was used for molecular docking, and the results were calculated. The docking results of the critical target proteins with the best binding ability to the compounds were shown by PyMOL software.

### Statistical analysis

All data were expressed as the mean ± standard deviation (Mean ± S.D). Statistical graphs were performed using GraphPad Prism 8.0.2 software (San Diego, CA, United States). *P* < 0.001, *p* < 0.01 or *p* < 0.05 were considered being significant.

## Results

### Chemical constituents of fresh ginseng paste and acquisition of active constituents of “RG-ZJ” herb pair

**FIGURE 1:**
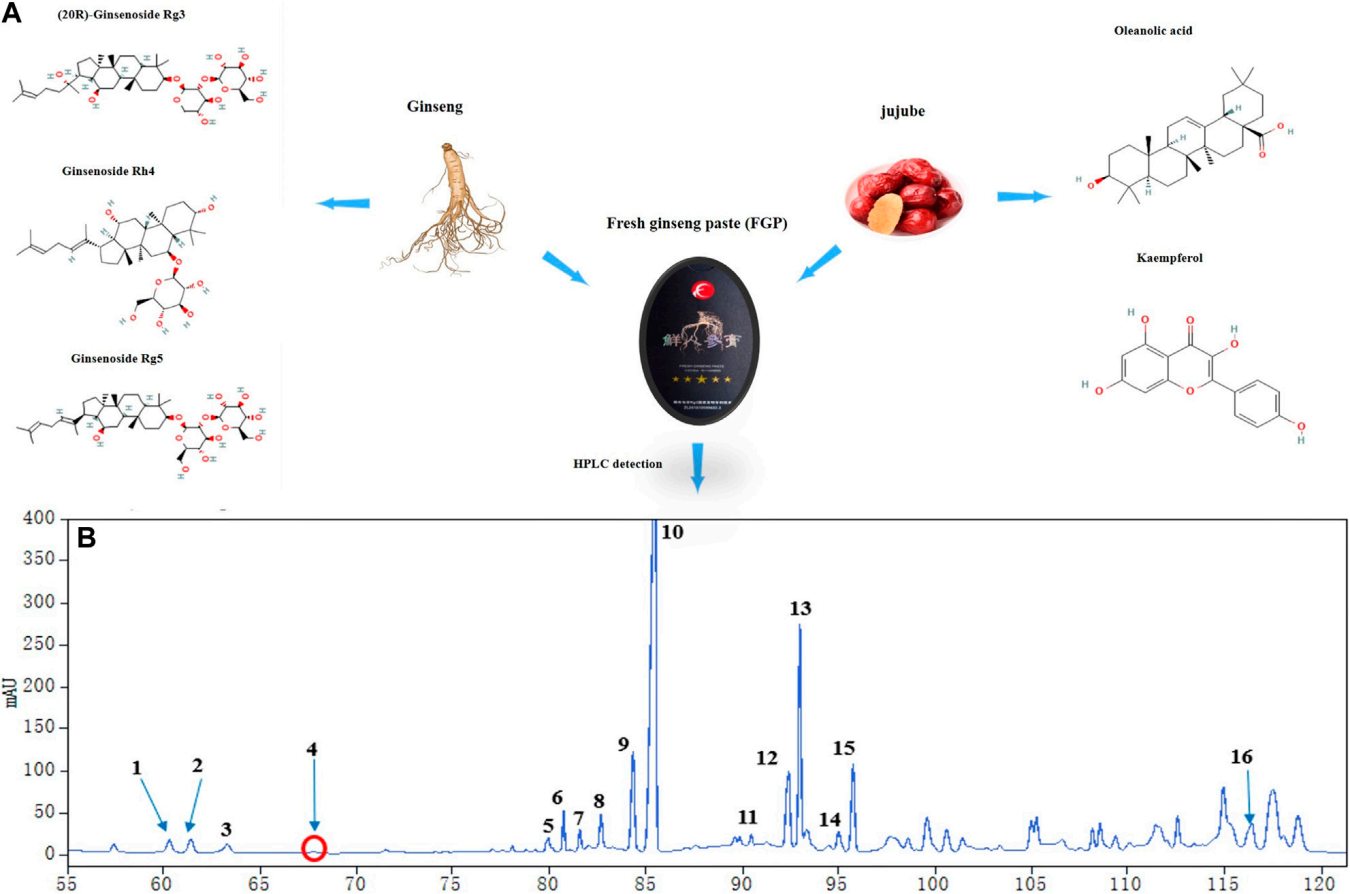
Analysis of principal components in FGP. Main components of Fresh ginseng paste (FGP) **(A)**; Identification of the main components in FGP by HPLC analysis **(B)**; 1. (S)-Rg2; 2. (S)-Rh1; 3. (R)-Rh1; 4. F1; 5. Rg6; 6. F4; 7. Rk3; 8. Rh4; 9. (S)-Rg3; 10. (R)-Rg3; 11. CK; 12. Rk1; 13. Rg5; 14. (S)-Rh2; 15. (R)-Rh2; 16. PPD.

As shown in Table 2, the “RG-ZJ” active ingredients data were screened according to the TCMSP database and related literature. The results showed that 46 effective compounds and corresponding gene targets were obtained (Figure 1), and HPLC identified saponins in FGP. Quantitative analysis of saponins in FGP was determined as follows: 1. (S)-Rg2:1.76%; 2. (S)-Rh1:1.88%; 3. (R)-Rh1:1.59%; 4. F1:0.06%; 5. Rg6:1.76%; 6. F4:2.79%; 7. Rk3:2.09%; 8. Rh4:3.06%; 9. (S)-Rg3:8.16%; 10. (R)-Rg3:36.15%; 11. CK:0.66%; 12. Rk1:6.56%; 13. Rg5:17.33%; 14. (S)-Rh2:1.91%; 15. (R)-Rh2:7.09%; 16. PPD:3.00%. The total saponin content in FGP was 0.883 g/100 g FGP (Table 2).

**TABLE 2:** Content of ginsenosides in FGP.

Ingredients	Content (mg/g)	Ingredients	Content (mg/g)
(S)-Rg2	0.321	Rg6	0.332
(S)-Rh2	0.345	F4	0.525
(R)-Rh2	0.299	Rk3	0.395
F1	0.012	Rh4	0.576
(S)-Rg3	1.536	Rg5	3.263
(R)-Rg2	6.807	(S)-Rh2	0.360
CK	0.125	(R)-Rh2	1.335
Rk1	1.236	PPD	0.565

### Network construction and enrichment analysis of “RG-ZJ” herb pair and HUA-related targets

**FIGURE 2:**
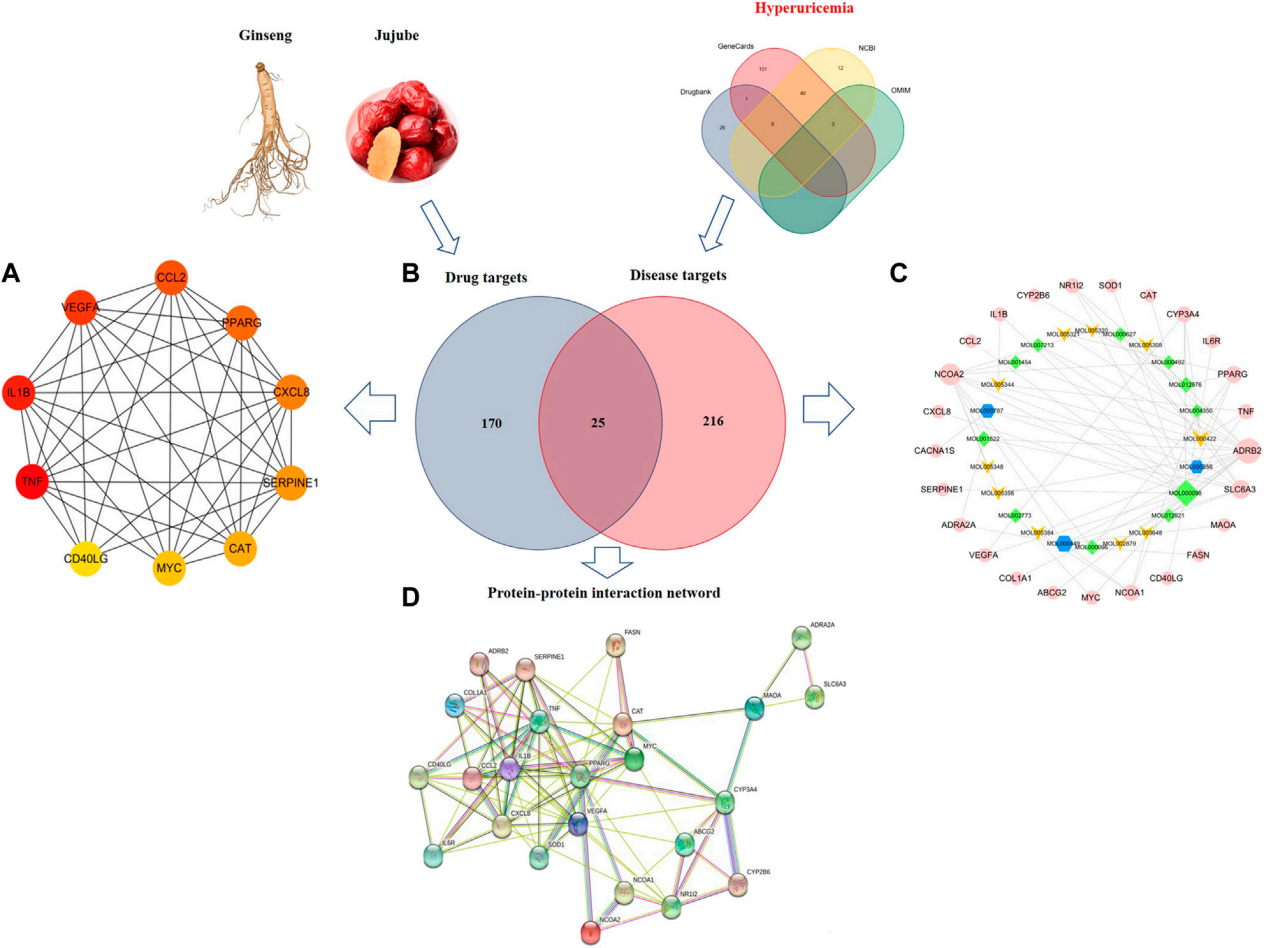
Visualization of the interaction targets between “RG-ZJ” components and the HUA disease diagram. Screened core targets **(A)**; Venn diagram showing the numbers of the overlapped and specific targets among the RG- ZJ herb pair (blue circle) and HUA (red circle) **(B)**; Compound-target-pathway network of the “RG- ZJ” herb pair against HUA. The light green and yellow nodes are active ingredients of ZJ and RG, respectively. The pink nodes is the potential targets **(C)**; Construction of protein interaction network **(D)**.

**FIGURE 3:**
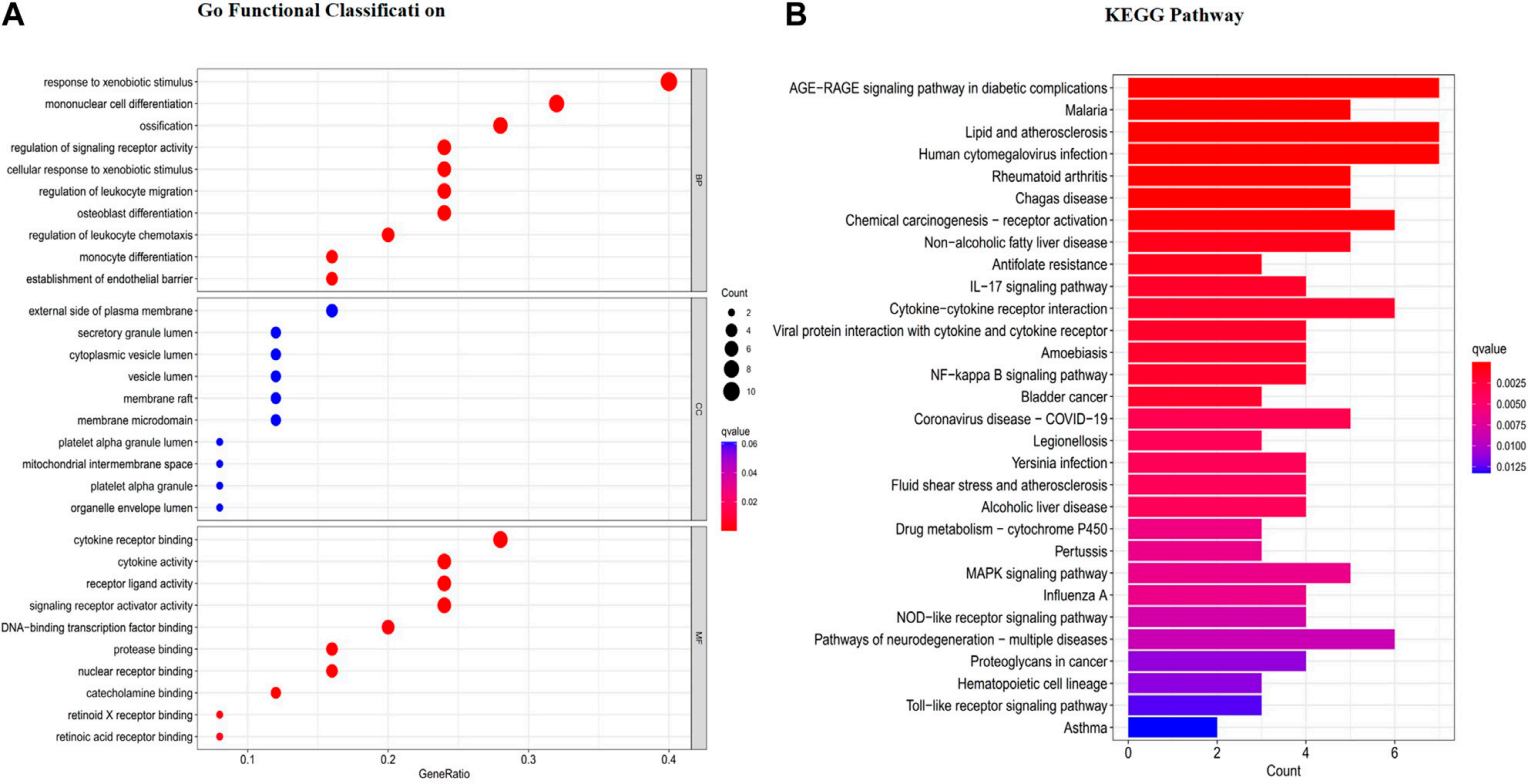
GO enrichment **(A)** and KEGG pathway analysis **(B)** of the HUA targets of the “RG-ZJ” herb pair.

We associated drug targets with disease genes, obtained 25 targets of 12 active components in Figures 2B,C, and further constructed a regulatory network diagram of active ingredients and related targets in “RG- ZJ.” There were 49 nodes and 73 edges. As shown in Figure 3A, we obtained 1140 biological processes (BP), 147 cellular components (CC), and 69 molecular functions (MF) by GO enrichment analysis, involving responses to heterogeneous stimuli, regulation of leukocyte chemotaxis, osteoblast differentiation, regulation of leukocyte migration, and mitochondrial membrane gap, platelet alpha particle lumen, and membrane microregions. We obtained 64 related signaling pathways, including Toll-like receptors and IL-17, and MAPK signaling pathways through KEGG enrichment analysis (Figure 3B).

### Effect of fresh ginseng paste on biochemical indicators in Hyperuricemia mice

**FIGURE 4:**
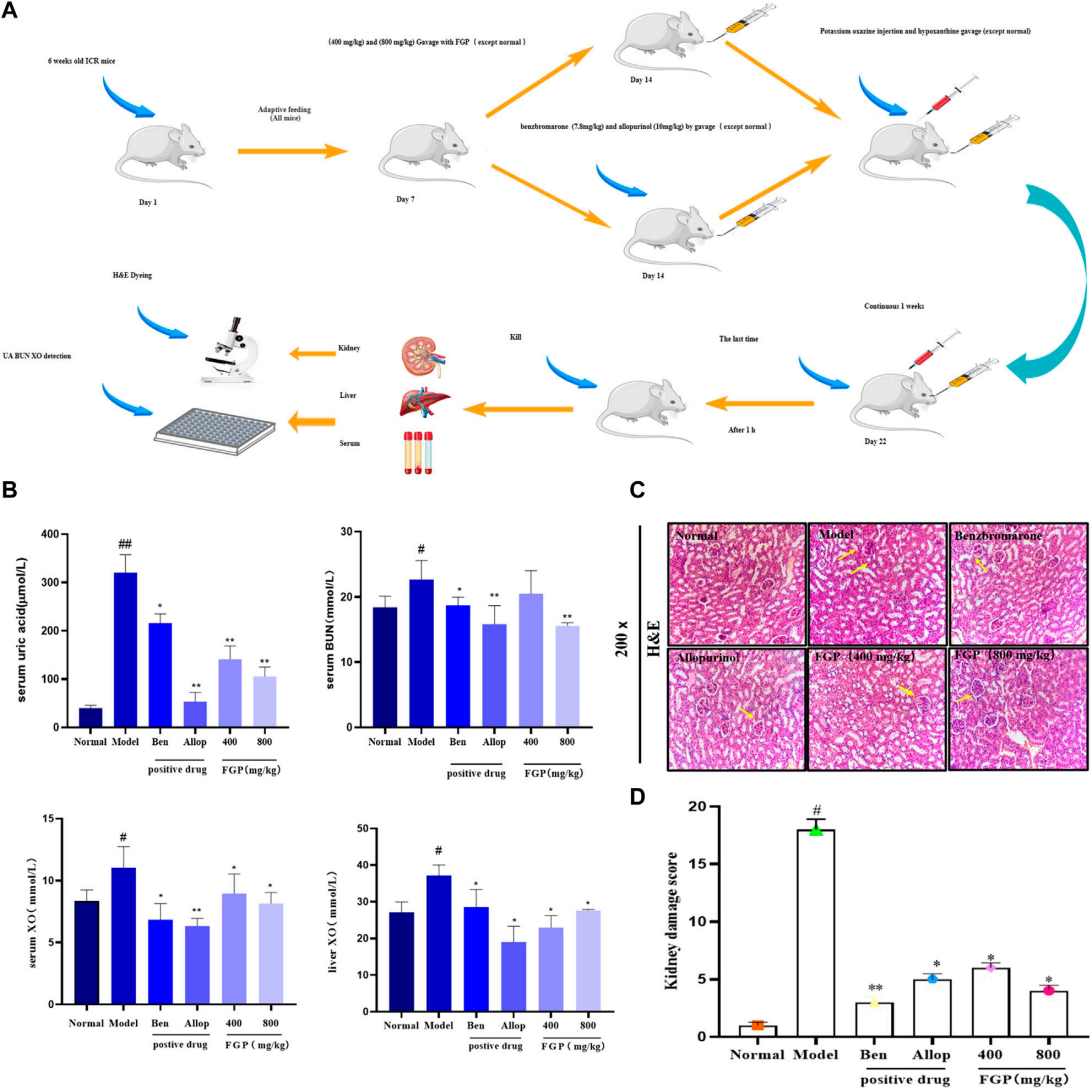
Effects on related indexes in HUA mice. Experimental animals and design process **(A)**. Effects of FGP on UA, BUN levels in the serum in mice and effects of FGP on XO activities in the liver and serum in mice **(B)**; Histological examination of the kidney (200 ×) **(C)**; Pathological changes of kidney and Ridit analysis **(D)**. Data are expressed as the mean ± SD (*n* = 8). ^#^ *p* < 0.05, ^#^^#^ *p* < 0.01 vs. normal group; *p < 0.05, **p < 0.01 or ***p < 0.001 vs. HUA group.

As shown in Figure 4, compared with the normal group, the levels of UA, BUN and XO after PO (100 mg/kg) and HX (500 mg/kg) administration were significantly increased (*p* < 0.05) (*p* < 0.05). It is indicated that the HUA model was successfully established. After administration of Ben and All, the UA level in mice was significantly decreased (*p* < 0.05). In addition, the levels of UA, BUN, and XO in the 400 mg/kg and 800 mg/kg of FGP were significantly lower than this in the HUA group (*p* < 0.05). In particular, the level of UA in the serum of mice was significantly reduced (*p* < 0.01).

### Effect of fresh ginseng paste on histopathological alterations in Hyperuricemia mice

The results showed that compared with the normal group, the renal coefficient of the model group was significantly increased (*p* < 0.05), which was related to renal edema caused by HUA (Table 3). However, both positive drugs and FGP treatment attenuated this change. In addition, the H&E staining results in Figures 4E,F showed that compared with the normal group, the number of glomeruli in the model group was significantly reduced (*p* < 0.05), and there were local edema, glomerular ablation, and unclear boundary. However, FGP effectively improved this pathological state, reduced glomerular ablation, and increased the number of glomeruli. The above results show that FGP could promote the excretion of UA and avoid renal edema.

**TABLE 3:** Effects of FGP on organ index of HUA mice.

Groups	Dosage (mg/kg)	Weights (g)	Kidney index (%)
Normal	—	38.16 ± 3.04	1.43 ± 0.16
Model	—	38.14 ± 2.69	1.76 ± 0.13#
Benzbromarone	7.8	36.86 ± 2.41	1.48 ± 0.14*
Allopurinol	5	37.52 ± 2.55	1.54 ± 0.19
FGP-H	400	37.73 ± 2.35	1.5 ± 0.15*
FGP-L	800	37.88 ± 1.87	1.49 ± 0.13*

Note: ^#^ *p* < 0.05, ^##^ *p* < 0.01 vs. normal group; *p < 0.05, **p < 0.01 vs. HUA, group.

### Molecular docking validation of core targets and active compounds

**FIGURE 5:**
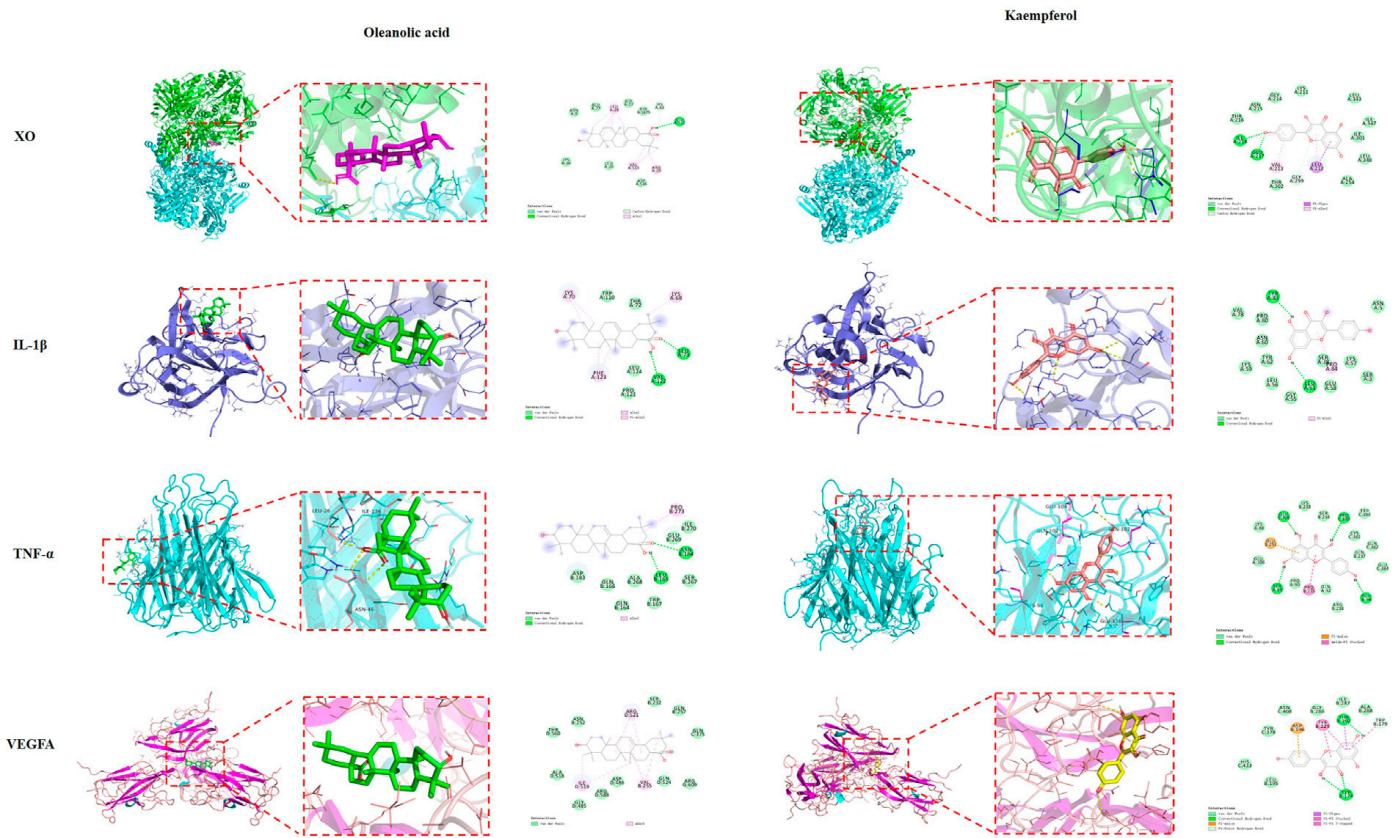
Molecular docking of oleanolic acid and kaempferol with TNF-α, IL-1B, VEGFA, and XO, respectively.

**FIGURE 6:**
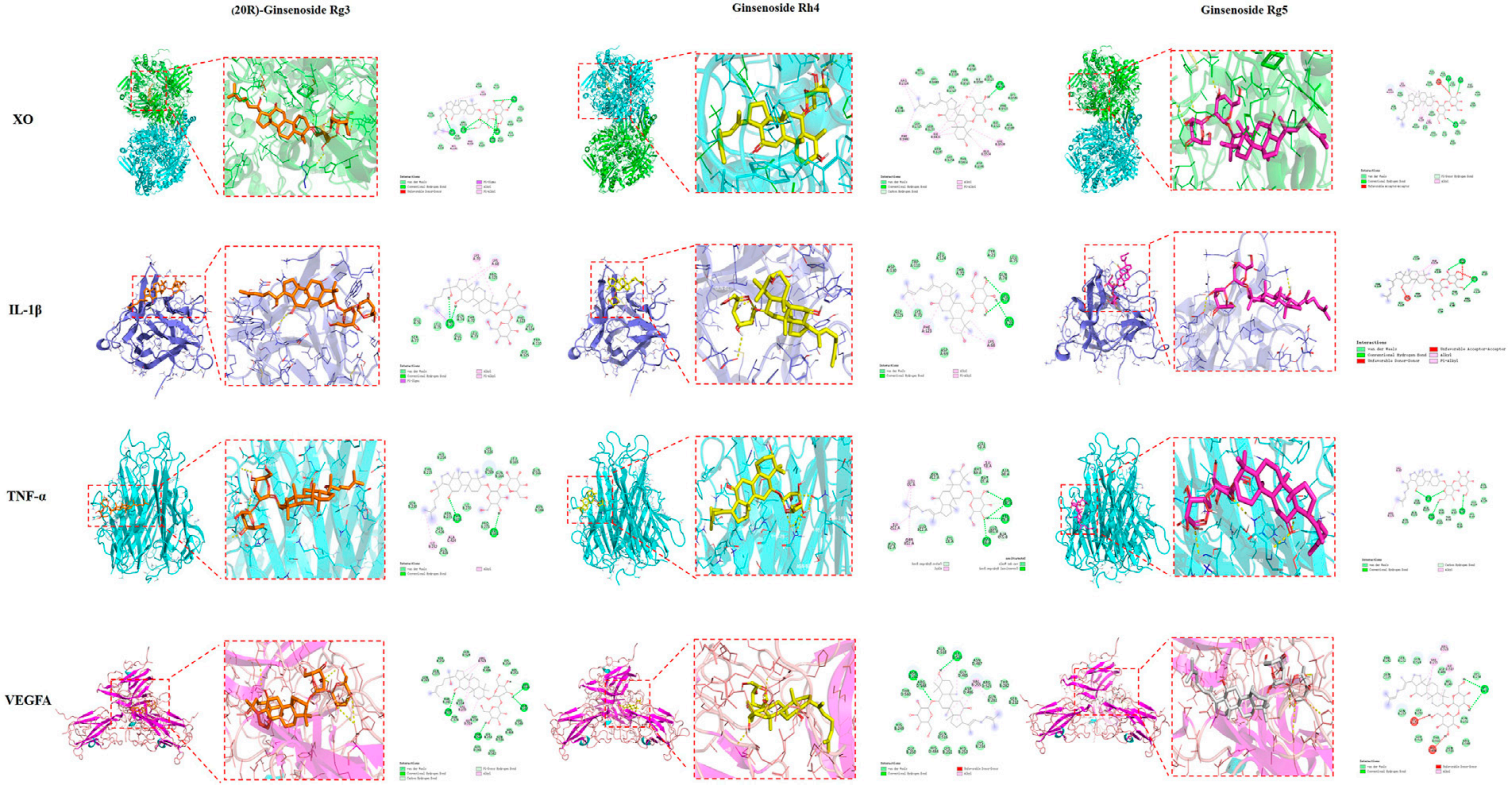
Molecular docking of 20(R)-ginsenoside Rg3, ginsenoside Rg5, and ginsenoside Rh4 with TNF-α, IL-1βVEGFA, and XO, respectively.

Molecular docking was applied to analyze the binding of the critical target TNF-α, IL-1β, VEGFA and XO with five main active compounds in FGP. The binding energy between the above compounds and the target was lower than −5.0 kJ/mol (Table 4). As shown in Figures 5, 6, the key targets TNF-α, IL-1β, VEGFA, and XO were stably combined with five compounds, including (20R)-ginsenoside Rg3, ginsenoside Rg5, ginsenoside Rh4, oleanolic acid, and kaempferol. The results showed that the above compounds of FGP could affect the critical targets of HUA. It is worth noting that XO has been studied as an inhibitory target for UA reduction in many natural products. Interestingly, the compounds in our FGP had excellent binding activity to XO (Binding energies were less than −7.0 kJ/mol).

**TABLE 4:** Docking of core targets with compounds in FGP.

Herbs	Ingredients	Hub gene binding energy (kcal/mol)			
ZJ		XO	IL-1β	TNF-α	VEGFA
	Oleanolic acid	−9.4	−7.5	−6.9	−8.7
	Kaempferol	−9.5	−7.2	−8.6	−8.2
RG	(20R)-Ginsenoside Rg3	−9.8	−7.0	−6.4	−9.1
	Ginsenoside Rh4	−9.2	−7.2	−6.9	−8.9
	Ginsenoside Rg5	−9.5	−7.4	−7.8	−9.0

## Discussion

Excessive UA level is considered the main cause of HUA (Zhang et al., 2018). Renal excretion accounts for 70% of total UA elimination, with the remaining 30% eliminated in the intestine. Renal UA underexcretion is the leading cause of HUA, found in approximately 90% of HUA patients (Lee et al., 2018). Both Radix ginseng (RG) and Ziziphus jujube (ZJ) have the effect of invigorating the kidney and enhancing metabolism (Rekha et al., 2021). Therefore, this study explored the potential mechanism of the “RG-ZJ” drug in the treatment of HUA through network pharmacology, molecular docking, and animal experiments.

At present, the research on the analysis of active compounds obtained from traditional Chinese medicine (TCM) and disease targets through network pharmacology is increasing, especially for herb pairs. The present study explored potential targets in the “RG-ZJ” herb pair for improving HUA through network pharmacology (Lu et al., 2020). The results showed that the “RG-ZJ” herb pair could improve HUA through CCL2, TNF-α, IL-1β, and VEGFA. Previous studies have analyzed the potential mechanism of Plantain in the treatment of HUA by network pharmacology and found that Plantain can affect HUA by interacting with MAPK1, TNF-α, and other targets (Pei et al., 2020). At the same time, some studies have predicted the targets of practical components in the “Gardenia-Poria” couplet on HUA, and the results show that the core targets of its role include IL-1β and IL-6 (Liu et al., 2021). In addition, previous studies found that baicalin and baicalein could improve HUA by down-regulating TLRs, NLRP3, and MAPK through network pharmacology analysis (Huilong et al., 2021). Combined with the above results, it can be found that the key pathways affecting HUA are mainly concentrated on the IL-17 signaling pathway and MAPK signaling pathway, which is consistent with the results of this study. Our prediction results were in line with previous reports and confirmed the reliability of the prediction on effective compounds of “RG-ZJ” against HUA. Besides, KEGG results also suggested that “RG-ZJ” might reduce the inflammatory response in HUA by affecting the TNF-α signaling pathway, IL-17 signaling pathway, and MAPK signaling pathway. The results are also consistent with previous studies.

The main role in the formation of UA is purines, and the accumulation of purines in the body is the main reason for the production of HUA. XO is the critical enzyme in the last step of UA synthesis, which catalyzes the oxidation of hypoxanthine to xanthine and then to UA, or directly to UA (Furuhashi et al., 2018; Kawachi et al., 2021). Several studies have shown that flavonoids (Scheepers et al., 2019) and saponins (Xu et al., 2022) isolated from TCM can reduce UA by reducing the content of XO in the liver while enhancing renal excretion of UA. Meanwhile, previous reports have shown that Urtica extracts can reduce serum UA levels by improving renal metabolism (Liu et al., 2020). Related research found that *Hedyotis diffusa* extract could improve HUA symptoms by reducing UA and BUN levels in serum (Zhang et al., 2021). In this study, animal experiments found that FGP can significantly reduce the levels of UA, BUN, and XO in HUA mice and improve the renal injury caused by HUA. These results are consistent with the above research results.

Due to the limitations of network pharmacology, the screening of drug components and disease targets is not comprehensive. Therefore, based on network pharmacology, this study screened five compounds that might play a role in FGP. Three core targets are related to HUA, TNF, IL-1β, VEGFA, and XO, through a large number of literature and previous research accumulation. Through molecular docking technology, the above compounds were docked with the target. The results showed that the above compounds had a strong binding ability with targets, which further proved the possibility of FGP improving HUA and preliminarily revealed the possible mechanism of action. However, there were still some shortcomings in this research. Since the pathological development of HUA involves complex pathological processes, the mechanism predicted above of “RG-ZJ” in treating HUA still needs to be supplemented by *in vivo* and *in vitro* experiments. In the future, we will continue to explore the mechanisms of “RG-ZJ” practical components in the treatment of HUA diseases in the hope of providing new insights for clinical drug development.

## Conclusion

In summary, firstly we analyzed the target of interaction between “RG-ZJ” couplet medicines and HUA through network pharmacology. Then through molecular docking and animal experiments, the improvement effect of potential functional food FGP processed by “RG-ZJ” on HUA was revealed, and the possible effect mechanism was preliminarily explored, providing a theoretical basis for the further development of ginseng products.

## Data Availability

The original contributions presented in the study are included in the article/supplementary material, further inquiries can be directed to the corresponding author.

## Abbreviations

CP, Cisplatin; ER, Endoplasmic reticulum; FGP, Fresh Ginseng Paste; HUA, Hyperuricemia; BUN, serum urea nitrogen; UA, uric acid; XO, xanthine oxidase; XOIs, XO inhibitors; PPI, Protein-protein interaction; GO, gene ontology; PO, potassium oxazine; HX, hypoxanthine; Ben, benzbromarone; All, allopurinol.
